# Important Dates in the Chronology of Pharmacy

**Published:** 1913-09

**Authors:** 


IMPORTANT DATES IN THE CHRONOLOGY OF PHARMACY. John F. Llewellyn, Mexico, Mo.

B. C. 3500. Is date of the oldest prescription, written on Egyptian stone, which is in the Metropolitan Museum of Art, N. Y.

B. C. 2000. Chinese knew Rhubarb, Aconite, Bark of Pomegranate, Ergot of Rye, Camphor and Canella.

B. C. 2100. King Osimandias (Egypt) wrote above his library "the pharmacy of the soul," another rendering is "The office of remedies for diseases of the soul."

About this period pharmacy was separated from medicine in Egypt.

B. C. 1700 to 1400. There are three Egyptian papyrus, that are as much pharmacopoeias as medical treatises one mentions fifty vegetable substances, another sixty, that were used medicinally, besides those from animals and minerals.

Ointments, clysters and poultices are mentioned.

They appealed to the god who will "slay the slayer."

B. C. 1490 and 1000. The Bible mentions the art of the apothecary or perfumer. Moses probably had this from papyrus mentioned above which he is supposed to have studied.

Apothecary and perfumer were one in Egypt.

B. C. 1300. Chiron, Esculapius and his two sons, this date is an average of nine estimates.

B. C. 460-327. Hippocrates.

B. C. 132-63. Mithriadates and his Mithridate or Theriac.

A. D. 50. Celsus wrote an account of the medical system of his time.

A. D. 65. Pliny wrote a materia medica.

A. D. 100. Dioscerides wrote a treatise on materia medica and edited a pharmacopoeia.

A. D. 117. In Baden near Zurich there were found Roman ruins containing medical pharmaceutical and surgical appliances, medical spoons in bone and silver, measuring vessels, jars and pots, some containing traces of ointments; the latest coins found were those of Hadrian.

A. D. 130. Galen laid the foundation for galenicals.

A. D. 650. The University of Salerno early in the seventh century taught pharmacy and the separation of medicine and pharmacy.

A. D. 750. Early in the eighth century Al Mansur established a pharmacy.

A. D. 806. Arabs produced a pharmacopoeia and established apothecary shops.

A. D. 829. Monastery of St. Gall had plans for a hospital and pharmacy.

A.D . 857. Schools of pharmacy arose in the chief Moslem cities.

A. D. 949. Cordova made advancement in medicine greater than any since Galen.

A. D. 1050. Monte Cassino, near Naples, had a monastery hospital, infirmary and pharmacy.

A. D. 1145. St. Hildegarde prepared a materia medica.

A. D. 1225. St. Elizabeth of Hungary established a sisterhood to nurse the sick and had a sisterhood pharmacy.

A. D. 1241. Frederick Domkellar presented his apothecary shop to the monastery of St. Thomas.

A. D. 1250. Established a drug store, privileges protected by government in Germany and France.

A. D. 1307. In Ragusa, Dalmtia is now a San Franciscan pharmacy established in 1307.

A. D. 1535. Henry VIII amused himself making cramp rings, plasters and compounding medicines.

A. D. 1540. The citizens of London agreed to buy for St. Bartholomew's Hospital all manner of apothecary wares and all that was necessary for making salves and all other things touching physic or surgery.

A. D. 1606. Louis Hebert, apothecary, came from France, and in 1616 returned and brought out his family.

A. D. 1613. Besler, a pharmacist of Nuremberg, published a work on botany.

A. D. 1625. Dalmahoy kept a shop on Ludgate Hill, where he sold drugs, potions, electuaries, powders, sweetmeats, wares for the complexion, scented hair oil pomades, dentifrice, love charms, Italian masks to sleep in, spermaceti salts, scammony and squills.

A. D. 1698. An English physician reported Paris apothecary shops neat enough, if they were as well stored with medicines.

A. D. 1716. Douglas, an apothecary, was raised to the peerage.

A. D. 1732. Thomas Harwood of Boston wrote a treatise on pharmacy.

A. D. 1785. Stark's Pharmacy, London, established, yet in business.

A. D. 1851. Organization of the American Pharmaceutical Association.

—*Journal of the American Pharm. Asso.*

